# Mesenchymal Stromal Cell Secreted Sphingosine 1-Phosphate (S1P) Exerts a Stimulatory Effect on Skeletal Myoblast Proliferation

**DOI:** 10.1371/journal.pone.0108662

**Published:** 2014-09-29

**Authors:** Chiara Sassoli, Alessia Frati, Alessia Tani, Giulia Anderloni, Federica Pierucci, Francesca Matteini, Flaminia Chellini, Sandra Zecchi Orlandini, Lucia Formigli, Elisabetta Meacci

**Affiliations:** 1 Department of Experimental and Clinical Medicine - Section of Anatomy and Histology, University of Florence, Florence, Italy; 2 Department of Biomedical, Experimental and Clinical Sciences “Mario Serio” - Unit of Biochemical Sciences and Molecular Biology, University of Florence, Florence, Italy; Stem Cell Research Institute, Belgium

## Abstract

Bone-marrow-derived mesenchymal stromal cells (MSCs) have the potential to significantly contribute to skeletal muscle healing through the secretion of paracrine factors that support proliferation and enhance participation of the endogenous muscle stem cells in the process of repair/regeneration. However, MSC-derived trophic molecules have been poorly characterized. The aim of this study was to investigate paracrine signaling effects of MSCs on skeletal myoblasts. It was found, using a biochemical and morphological approach that sphingosine 1-phosphate (S1P), a natural bioactive lipid exerting a broad range of muscle cell responses, is secreted by MSCs and represents an important factor by which these cells exert their stimulatory effects on C2C12 myoblast and satellite cell proliferation. Indeed, exposure to conditioned medium obtained from MSCs cultured in the presence of the selective sphingosine kinase inhibitor (iSK), blocked increased cell proliferation caused by the conditioned medium from untreated MSCs, and the addition of exogenous S1P in the conditioned medium from MSCs pre-treated with iSK further increased myoblast proliferation. Finally, we also demonstrated that the myoblast response to MSC-secreted vascular endothelial growth factor (VEGF) involves the release of S1P from C2C12 cells. Our data may have important implications in the optimization of cell-based strategies to promote skeletal muscle regeneration.

## Introduction

Bone-marrow-derived mesenchymal stromal cells (MSCs) are currently considered among the best candidates in the field of regenerative medicine. Numerous experimental studies have shown the beneficial effects of MSC transplantation in tissue and organ repair/regeneration and clinical trials are actually ongoing [1]–[6]. A large body of experimental evidence has shown that transplantation of MSCs in animal models of muscle injury and disease has great therapeutic potential [7]–[10]. Indeed, the systemic or local administration of MSCs into skeletal muscles subjected to traumatic injuries such as laceration [7], crush or resection [9]–[11], or cardiotoxin injection [8], [12], has been demonstrated to contribute to myofiber formation and to the functional recovery of the muscle tissue. A considerable increase in the capillary density and collateral perfusion, associated with a reduction of myofiber atrophy and disarray, has also been observed in ischemic skeletal muscles transplanted with MSCs [13], [14]. Moreover, there are studies showing that the injection of MSCs into dystrophic muscles is capable to restore dystrophin expression [12], [15], [16], attenuate the oxidative stress [17] and improve the contractile function [15]. In most of the reported studies, the therapeutic effects of MSCs do not seem to be attributed to their differentiation into resident cell types, but rather to their ability to release paracrine factors capable of improving the host tissue microenvironment and stimulate the endogenous mechanisms of tissue repair [2], [18], [19]. Therefore, the identification of stem cell secreted proteins, as well as of their downstream signaling pathways, is of great biological importance for extending the studies and ameliorating the results obtained after MSC transplantation. In this context, we have recently demonstrated that MSCs stimulate skeletal myoblast proliferation and differentiation through the release of vascular endothelial growth factor (VEGF) [20]. Indeed, MSCs release VEGF and the treatment with the selective pharmacological VEGF receptor inhibitor, KRN633, results in a marked attenuation of the receptor activation and in the inhibition of C2C12 cell proliferation induced by MSC-conditioned medium.

Sphingosine 1-phosphate (S1P) is a natural potent and multifunctional phospholipid mainly released into circulation by activated platelets and erythrocytes, but also by different cell types such as cerebellar astrocytes and glioma cells [21]–[24]. S1P is reported to exert a broad range of biological responses in many cell types including skeletal muscle cells [25]–[32]. Most of the known actions of S1P are mediated by a family of five specific G protein-coupled receptors (S1P_1–5_) which are present in muscle cells; their activation by S1P has been shown to promote skeletal myoblast proliferation, differentiation and survival [24], [26], [32], [33]. In particular, we have recently demonstrated that exogenous S1P attenuates the muscle damage induced by eccentric contraction, protecting the muscle fibers from apoptosis and preserving satellite cell viability and renewal [31].

Because of the proven therapeutic effects of S1P and MSCs in skeletal muscle healing, in the current study we evaluated whether MSCs could mediate the stimulation of skeletal myoblast proliferation through the release of S1P in order to extend and better define the molecular mechanisms underlying the paracrine interaction between the two cell types. Here, we demonstrated for the first time, that MSCs produce and secrete a large amount of S1P in the culture medium and that this sphingolipid is required for MSC-mediated effects on muscle C2C12 and satellite cell proliferation. Because it has been suggested that VEGF may crosstalk with S1P pathway [34]–[36], we also investigated the reciprocal interaction between these factors in stimulating myoblast proliferation. Our data may contribute to define the mechanisms by which donor MSCs contribute to muscle repair and regeneration and to optimize cell therapy for muscle disorders.

## Materials and Methods

### Ethics statement

All animal manipulations were carried out according to the European Community guidelines for animal care (DL 116/92, application of the European Communities Council Directive of 24 November 1986; 86/609/EEC) and approved by the Committee for Animal Care and Experimental Use of the University of Florence. The ethical policy of the University of Florence conforms to the Guide for the care and use of laboratory animals of the U.S. National Institutes of Health (NIH Publication No. 85-23, revised 1996; University of Florence assurance No. A5278-01). The protocols were communicated to local authorities and to Italian Ministry of the Health; according to the Italian law (Art.7/D.lgs 116/92) such procedure doesn't require Ministry authorization. The animals were housed with free access to food and water and maintained on a 12 h light/dark cycle at 22°C room temperature (RT). All efforts were made to minimize the animal suffering and the number of animals sacrificed. Animals were killed by decapitation.

### Cell culture and treatments

*Mouse bone marrow mesenchymal stromal cells* (MSCs) were isolated from femura and tibiae of male C2F1 mice, following the Dobson's procedure [37], expanded in vitro in low glucose Dulbecco's modified Eagle's medium (DMEM) supplemented with 1% L-glutamine, 1% penicillin/streptomycin, 25 mM HEPES, pyruvate and 20% fetal bovine serum (FBS; Sigma, Milan, Italy), and characterized as reported previously [20]. In some experiments, these cells were cultured at 37°C in a humidified atmosphere of 5% CO_2_, in C2C12 myoblast differentiation medium (DM) or in muscle satellite culture medium (PM) for 48 h [19] in the absence or in the presence of sphingosine kinase inhibitor (iSK, 5 µM, Tocris, Bristol, UK) [38], and the conditioned culture medium (MSC-DM, MSC-DMiSK, MSC-PM and MSC-PMiSK) was harvested and used for culturing C2C12 myoblasts or satellite cells, in order to assess MSC paracrine effects.

*Murine C2C12 skeletal myoblasts* obtained from American Type Culture Collection (ATCC, Manassas, VA, USA), were grown in DMEM supplemented with 10% FBS, 1% L-glutamine, 1% penicillin/streptomycin (Sigma) at 37°C in a humidified atmosphere of 5% CO_2_ till reaching 60–80% confluence and shifted in differentiation medium (DM), containing 2% horse serum (HS, Sigma).

In order to assess the paracrine effect of MSCs, C2C12 cells were exposed for 24 h to conditioned medium obtained from MSCs cultured in myoblast DM for 48 h in the absence (MSC-DM) or in the presence of 5 µM iSK (MSC-DMiSK). In other set of experiments, the cells were exposed to sphingosine 1-phosphate (S1P, 1 µM, Calbiochem, San Diego, CA, USA) for 24 h. In some experiments C2C12 cells were incubated for 24 h in conditioned medium obtained by culturing C2C12 myoblasts in DM in the presence or in the absence of iSK for 48 h.

In order to assess the role of S1P-released by MSCs in VEGF signaling, C2C12 cells were exposed to MSC-DM or MSC-DMiSK and treated or not with an ATP competitive inhibitor of vascular endothelial growth factor receptor (VEGFR) tyrosine kinase activity (KRN633, 170 nM, Santa Cruz Biotechnology, Santa Cruz, CA, USA) in the absence or in the presence of 1 µM S1P.

In other experiments, with the aim to assess the role of SphK/S1P axis in myoblast proliferation induced by MSC-released paracrine factors including VEGF, C2C12 myoblasts were cultured in MSC-DM and treated or not with MK571 (10–20 µM, Tocris, Bristol, UK) an inhibitor of ABCC1 (MRP1) transporter, known to regulate S1P export in some cells [39] or with 5 µM iSK.

To confirm the crosstalk between SphK/S1P axis on VEGF-induced signaling, C2C12 myoblasts in DM were treated or not with 10–20 µM MK571 or 5 µM iSK for 24 h in the absence or in the presence of soluble VEGF (2 ng/ml, Sigma) as reported previously [20].

*Satellite cells* were isolated from single myofibers of *Extensor Digitorum Longus* (EDL) muscles carefully removed from anesthetized (with 50 µg/g zolazepam tiletamine) young adult male Swiss mice (25–30 g) essentially as described previously [19]. Briefly, EDL muscles, soon after isolation, were digested in 0.2% collagenase type I in DMEM (Sigma) and then transferred to a Petri dish containing serum-free DMEM, in which single muscle fibers were liberated from the muscles, by means a gentle mechanical trituration with a Pasteur pipette. The intact, viable muscle fibers were collected and subsequently cultured individually in Matrigel (BD Biosciences, San Jose, CA, USA) treated 24 well plates, at 37°C in a humidified atmosphere of 5% CO_2_, in satellite cell proliferation medium (PM) containing DMEM, 20% FBS, 10% HS (Sigma), 0.5% chicken embryo extract (Sera Laboratories International Ltd, Horsted Keynes, UK) plus 1% L-glutamine and 1% penicillin/streptomycin (Sigma) for 48 h. After that, the myofibers were removed and the derived satellite cells were expanded in culture in PM before be used for the experiments. The cells (P1) were cultured as follows: in PM in the absence (control) or in the presence of 1 µM S1P (Calbiochem) or with conditioned medium obtained from MSCs cultured in PM for 48 h in the absence (MSC-PM) or in the presence of 5 µM iSK (MSC-PMiSK) stimulated or not with 1 µM S1P for 24 h. Aliquots of cells at P1 culture passage were seeded on glass coverslips and assayed for Pax-7 and vimentin (fibroblast marker) immunophenotype by confocal microscopy to test the degree of purity of satellite cell cultures which usually was 80%.

### Sphingolipid metabolism

MSCs were plated on 35 mm dishes and cultured in cell culture medium as described above. At 80% confluence the cells were shifted in DMEM supplemented with 10% FBS and pulsed with [^3^H]Sphingosine (Sph) (0,6 µCi/ml) for 2 h. In other experiments MSCs were pulsed with [^3^H]serine (10 µCi/ml) and incubated with C2C12 cell DM or satellite cell PM in the absence or in the presence of 5 µM iSK. Cells and media were collected and total lipids extracted with chloroform/methanol (1∶2) as previously reported [23], [38], [40]. The phase containing [^3^H]Sphingolipids, including [^3^H]S1P or [^3^H]ceramide, was evaporated under nitrogen stream. The fractions containing cellular and extracellular [^3^H]S1P were collected and subjected to Thin Layer Chromatography (TLC) on silica gel plates (Merck, Darmstadt, Germany), using n-butanol/acetic acid/water (3∶1∶1, v/v/v) as solvent system. Cold S1P was added in lipid extraction mixture and used as internal standard. At the end of the TLC, the silica gel spot that contained [^3^H]S1P was scraped off and quantified by liquid scintillation counting [32], [38].

### Cell proliferation analysis

Cell counting, [^3^H]thymidine and EdU (5-ethynyl-2′-deoxyuridine) incorporation assays were used to determine C2C12 and satellite cell proliferation.

#### Cell counting

For cell counting the cells were incubated for 24 h in the specific medium (DM for C2C12 myoblasts and PM for satellite cells) or exposed to MSC-conditioned medium (C2C12 cells: MSC-DM and MSC-DMiSK; satellite cells: MSC-PM and MSC-PMiSK) in the presence or in the absence of the specific compounds as reported in the indicated experiments. After 24 h cells were trypsinized and counted after Trypan Blue incubation (0.2%, Sigma) in a Burker chamber by two different operators.

#### [^3^H]thymidine incorporation

[^3^H]thymidine incorporation assay was performed in the cells cultured as reported above; the cells were incubated for the last 2 h in the presence of methyl [^3^H]thymidine (1–2 µCi/ml) (Perkin Elmer, Monza, Italy) and then processed using trichloroacetic acid precipitation as reported previously [41]. The recovered radioactivity was measured in a beta counter (Beckman Coulter s.r.l., Milano, Italy).

#### EdU (5-ethynyl-2′-deoxyuridine) incorporation

EdU incorporation assay was performed by using the fluorescent Click-iT EdU Cell Proliferation Assay (Life Technologies, Grand Island, NY, USA) according to manufacturer's instructions. This assay is based on the incorporation of the pyrimidine analogue EdU in place of thymidine into newly synthesized DNA of replicating cells. Briefly, cells grown on glass coverslips were incubated in the presence of the provided solution of 10 µM EdU for 24 h. After that, the cells were washed with PBS, fixed with 0.5% buffered paraformaldehyde (PFA) for 10 min at RT, permeabilized with cold acetone for 3 min and then incubated with the Alexa Fluor 488 EdU detection solution for 30 min at RT, protected from light. After washing, the coverslips containing the labeled cells were mounted with an antifade mounting medium (Biomeda Gel mount, Electron Microscopy Sciences, Foster City, CA) and observed under a confocal Leica TCS SP5 microscope (Leica Microsystems, Mannheim, Germany) equipped with a HeNe/Ar laser source for fluorescence measurements and with differential interference contrast (DIC) optics. The number of the cells with EdU positive nuclei was evaluated in 10 random 200×200 µm square microscopic fields (63× ocular) in each cell preparation and expressed as percentage of the total cell number evaluated by labeling nuclei with PI (See confocal immunofluorescence paragraph).

### Confocal immunofluorescence

Both C2C12 myoblasts and satellite cells grown on glass coverslips were fixed with 0.5% PFA for 10 min at RT. After permeabilization with cold acetone for 3 min, the fixed cells were blocked with 0.5% bovine serum albumin (BSA; Sigma) and 3% glycerol in PBS for 20 min and then incubated with a rabbit polyclonal antibody anti-Ki67 (1∶100; Santa Cruz Biotechnology) overnight at 4°C. Fixed satellite cells were also incubated overnight at 4°C with a mouse monoclonal anti-Pax7 (1∶100; Santa Cruz Biotechnology) and with a goat polyclonal anti-vimentin (1∶40; Sigma). The immunoreactions were revealed by incubation with specific anti-rabbit or anti-goat Alexa Fluor 488-conjugated IgG or with anti-mouse Alexa Fluor 568-conjugated IgG (1∶200; Molecular Probes, Eugene, OR, USA) for 1 h at RT. In some experiments, counterstaining was performed with propidium iodide (PI, 1∶30; Molecular Probes) to reveal nuclei. Negative controls were carried out by replacing the primary antibody with non-immune serum; cross-reactivity of the secondary antibodies was tested in control experiments in which primary antibodies were omitted. After washing, the coverslips containing the immuno-labeled cells were mounted with an antifade mounting medium (Biomeda Gel mount, Electron Microscopy Sciences) and observed under a confocal Leica TCS SP5 microscope (Leica Microsystems). Observations were performed using a Leica Plan Apo 63×/1.43NA oil immersion objective. Series of optical sections (1024×1024 pixels each; pixel size 204.3 nm) 0.4 µm in thickness were taken through the depth of the cells at intervals of 0.4 µm. Images were then projected onto a single ‘extended focus’ image. The number of the cells with Ki67 positive nuclei was evaluated in 10 random 200×200 µm square microscopic fields (63× ocular) in each cell preparation and expressed as percentage of the total cell number.

### Statistical analysis

Data were reported as mean±SEM. Statistical significance was determined by one-way ANOVA and Newman-Keuls multiple comparison test or Student' *t* test. A p value≤0.05 was considered significant. Calculations were performed using GraphPad Prism software (GraphPad, San Diego, CA, USA).

## Results

### MSCs release S1P in the culture medium

**Figure 1:**
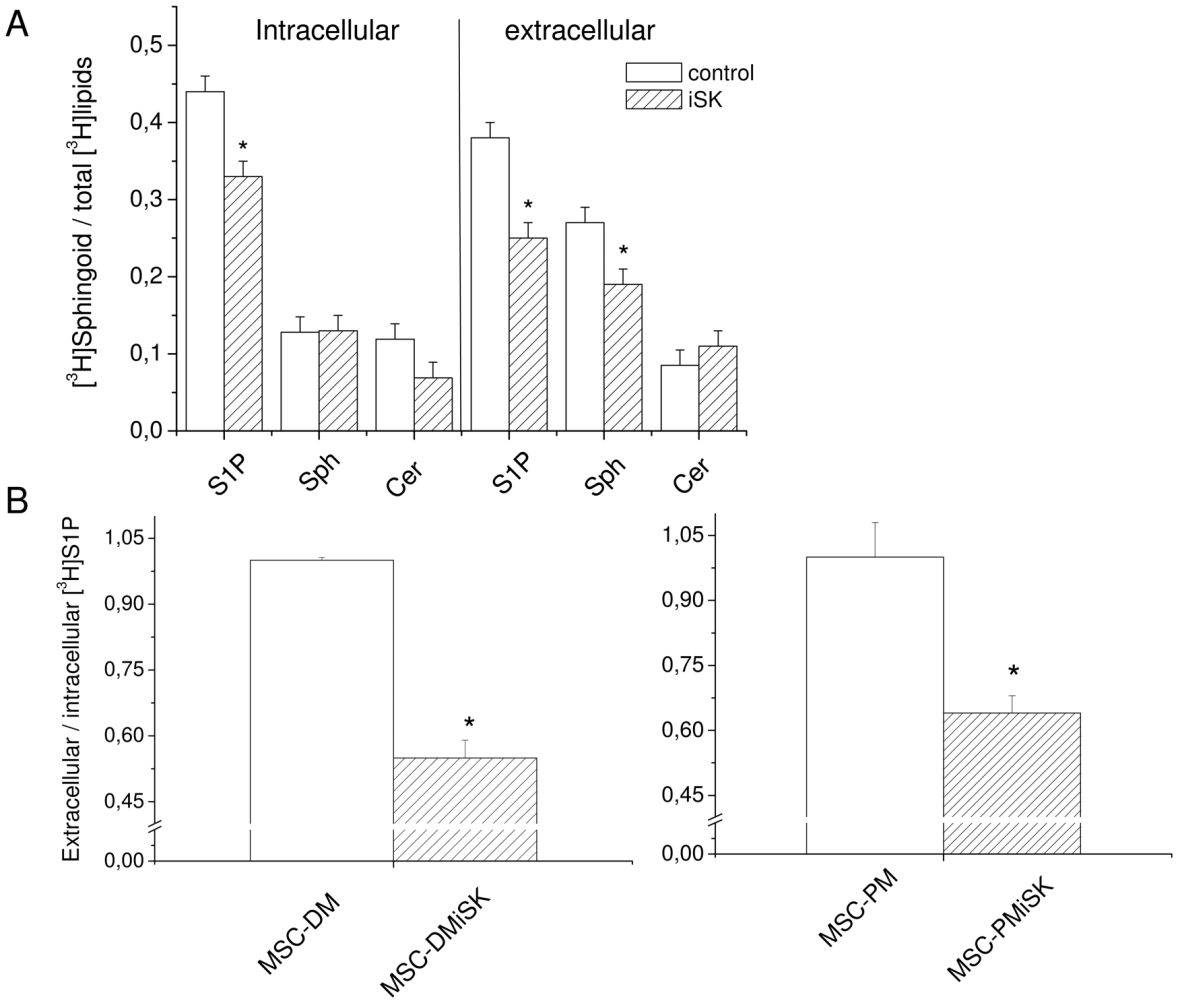
S1P content in [^3^H]sphingosine-pulsed MSCs. A) *[^3^H]sphingosine (Sph)-derived metabolites in MSCs*. MSCs were cultured in DMEM containing 10% FBS in the presence or in the absence of the selective sphingosine kinase inhibitor (5 µM, iSK) for 24 h and pulsed with [^3^H]Sph for 2 h. [^3^H]lipids (S1P; Sph; ceramide, Cer) were extracted from cells and conditioned medium and analyzed by TLC as described in *Materials and Methods* Section. B) *Extracellular/intracellular [^3^H]S1P ratio in MSCs*. MSCs were cultured in C2C12 myoblast differentiation medium (DM, DMEM containing 2% horse serum) or in satellite cell proliferation medium (PM) in the absence (MSC-DM, MSC-PM) or in the presence of 5 µM iSK (MSC-DMiSK, MSC-PMiSK) for 48 h and pulsed with [^3^H]Sph. [^3^H]lipids were extracted from the cells (intracellular) and the conditioned medium (extracellular) and processed as reported above. Note the significant reduction, in the content of [^3^H]S1P in the medium obtained from MSCs cultured in the presence of iSK. The results shown are mean±SEM of at least three independent experiments. Significance of difference (Student's t test): in A *p<0.05 *vs* control; in B *p<0.05 *vs* MSC-DM or MSC-PM.

Given that S1P has been reported to act both as an intracellular second messenger and as an extracellular mediator [42], we first evaluated by [^3^H]Sphingosine ([^3^H]Sph) pulse experiments, the ability of MSCs to release S1P into the extracellular medium. As shown in Fig. 1A, [^3^H]Sph incorporated into MSCs was metabolized mainly into [^3^H]S1P and [^3^H]ceramide (Cer), since the levels of other sphingoid molecules were not detectable in these experimental conditions (data not shown). [^3^H]S1P reduction was also detected at similar extent in the conditioned culture medium when MSCs were cultured both in C2C12 cell differentiation medium (MSC-DM) and satellite cell proliferation medium (MSC-PM) (Fig. 1B), suggesting the ability of MSCs to release the bioactive lipid. As shown in the same figure, the synthesis and extracellular release of [^3^H]S1P was dependent on sphingosine kinase (SphK) activity. Indeed, we observed a significant reduction of approximately 40% in the content of [^3^H]S1P in the conditioned medium obtained from MSCs cultured in DM or PM in the presence of the selective SphK inhibitor (5 µM; MSC-DMiSK and MSC-PMiSK). Similar results were obtained when MSCs were pulsed with [^3^H]serine and incubated with DM in the absence or in the presence of iSK (Table 1).

**Table 1:** S1P content in lysate and conditioned medium of [^3^H]serine pulsed MSCs.

	MSC-lysate *[^3^H]S1P/[^3^H]lipids*	MSC-conditioned medium *[^3^H]S1P/[^3^H]lipids*
***Control***	0,21±0,03 (100%)	0,1537±0,007 (100%)
**iSK**	0,13±0,02 (62±10%)	0,0926±0,01 (60±9,3%)

[^3^H]lipids were extracted by MSCs cultured in the presence or in the absence (control) of 5 µM sphingosine kinase inhibitor (iSK) and successively pulsed with [^3^H]serine for 2 h.

[^3^H]lipids were separated by TLC as described in Materials and Methods Section.

### S1P synthesis and release by MSCs affect skeletal muscle cell proliferation

**Figure 2:**
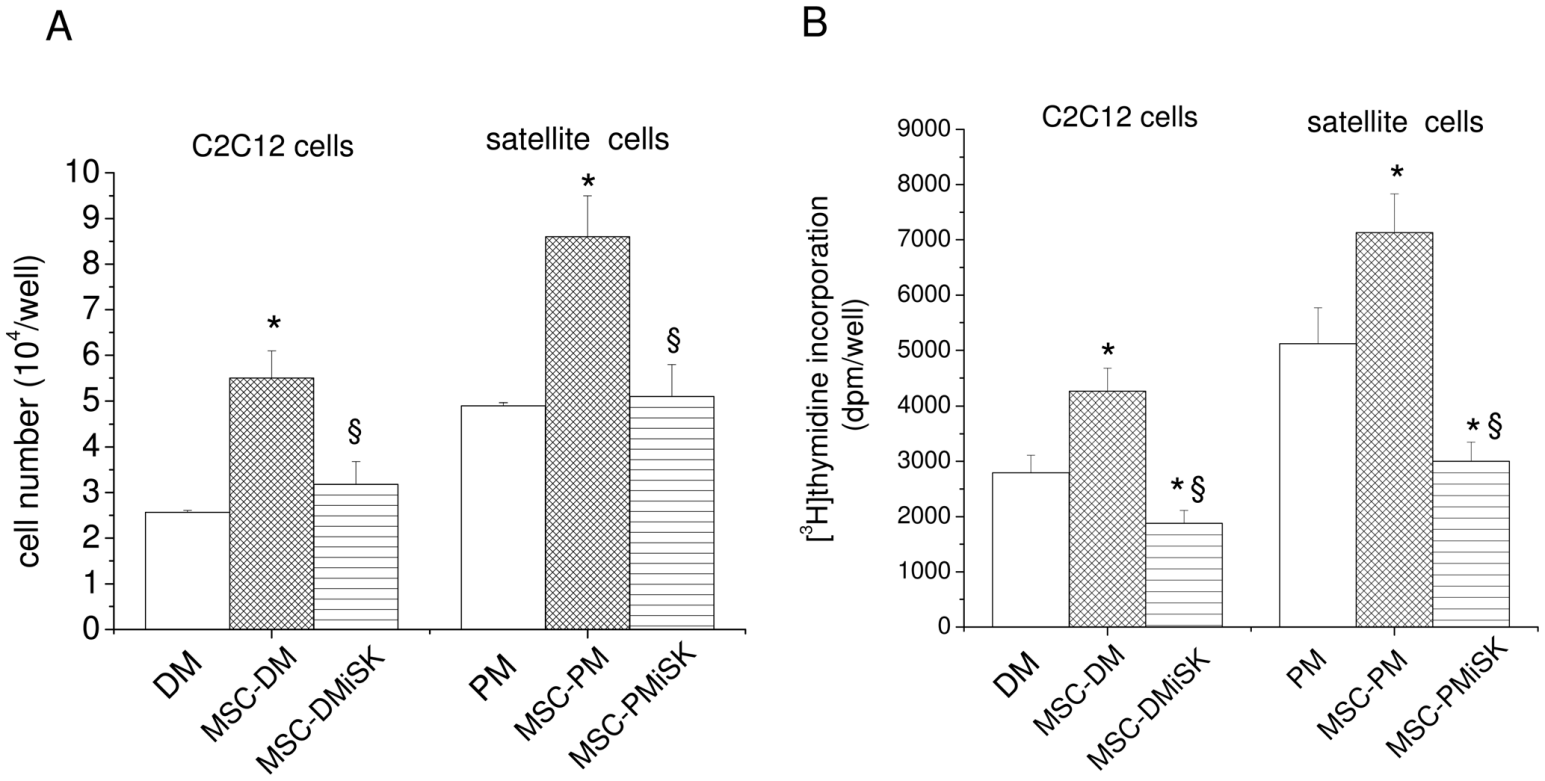
Effect of S1P secreted by MSCs on C2C12 and satellite cell proliferation: cell counting and [^3^H]thymidine incorporation. C2C12 or satellite cells isolated from single muscle fibers were cultured in differentiation medium (DM) or satellite cell proliferation medium (PM) or exposed to MSC-conditioned medium obtained by culturing MSCs for 48 h in DM in the absence (MSC-DM) or in the presence of 5 µM iSK (MSC-DMiSK), or in PM in the absence (MSC-PM) or in the presence of iSK (MSC-PMiSK), respectively. A) *Cell counting*. After 24 h of culture the cells were trypsinized and counted as described in Materials and Methods Section. B) *[^3^H]thymidine incorporation assay*. Cells were cultured for the last 2 h in the presence of methyl [^3^H]thymidine. C2C12 and satellite cells were processed as described in Materials and Methods Section and the recovered radioactivity measured in a beta counter. The results shown are mean±SEM of at least three independent experiments performed in duplicates. Significance of difference (Student's t test): *p<0.05 vs DM or PM; ^§^p<0.05 *vs* MSC-DM or MSC-PM.

We next evaluated whether the S1P released by MSCs could affect C2C12 and satellite cell proliferation. As reported in Fig. 2 the reduced content of S1P in the conditioned medium obtained from iSK-treated MSCs (MSC-DMiSK and MSC-PMiSK) attenuated C2C12 and satellite cell proliferation as evaluated by the cell number counting (Fig. 2A) and [^3^H]thymidine incorporation assay (Fig. 2B). In particular, we observed a decrease of 40–50% of both C2C12 and satellite cell number and DNA synthesis when the cells were exposed for 24 h to MSC-DMiSK and MSC-PMiSK, respectively, in comparison to specific control (MSC-DM and MSC-PM). Successively, we evaluated whether the potential residual active iSK in the conditioned medium could directly affect myoblast proliferation. We found no significant difference in C2C12 cell proliferation cultured for 24 h in the conditioned medium obtained from C2C12 cells incubated for 48 h in DM in the absence (C2C12-DM) or in the presence of iSK (C2C12-DMiSK). In particular, the number of proliferating C2C12 cells was 6,35±0,33 and 5,92 ±0.36 when the cells were exposed for 24 h to C2C12-DM and C2C12-DMiSK, respectively.

**Figure 3:**
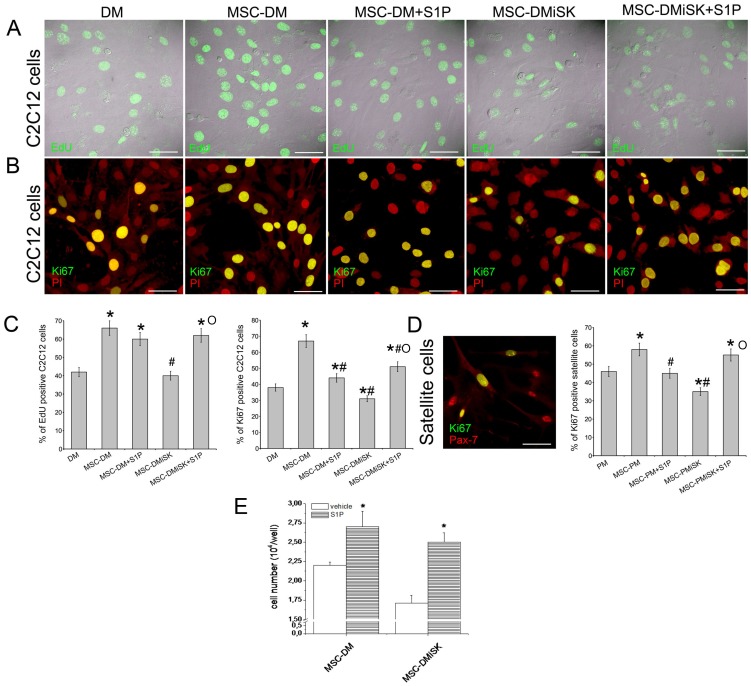
Effect of S1P secreted by MSCs and exogenous S1P on C2C12 and satellite cell proliferation: morphological evaluation and cell counting. A–D) *Morphological evaluation of C2C12 and satellite cell proliferation by EdU incorporation assay and Ki67 expression analysis*. A) Representative superimposed DIC and fluorescence images showing nuclear incorporation of EdU (green) in C2C12 cells cultured for 24 h in differentiation medium (DM) or exposed to MSC-conditioned medium obtained by culturing MSCs for 48 h in DM in the absence (MSC-DM) or in the presence of 5 µM iSK (MSC-DMiSK), and stimulated or not with 1 µM S1P (MSC-DM+S1P, MSC-DMiSK+S1P). Scale bar 50 µm. B) Representative confocal immunofluorescence images of C2C12 cells cultured in the indicated experimental conditions as in A, immunostained with antibodies against the nuclear protein Ki67 (green). Nuclei are counterstained in red with propidium iodide (PI). Yellow color indicates co-localization between red and green fluorescence signals. Scale bar 50 µm. C) Quantitative analysis of the percentage of EdU or Ki67 positive C2C12 cell nuclei expressed as percentage of the total nuclei number. D) Representative confocal immunofluorescence image of satellite cells cultured for 24 h in satellite cell proliferation medium (PM), fixed and immunostained with antibodies against Pax-7 (red) and Ki67 (green). Yellow color indicates co-localization between red and green fluorescence signals. Scale bar 50 µm. In the histogram the quantitative analysis of the percentage of Ki67 positive nuclei of satellite cells cultured for 24 h in PM or exposed to MSC-conditioned medium obtained by culturing MSCs for 48 h in PM in the absence (MSC-PM) or in the presence of 5 µM iSK (MSC-PMiSK), and stimulated or not with 1 µM S1P (MSC-PM+S1P, MSC-PMiSK+S1P). Data shown are mean±SEM and represent the results of at least three independent experiments with similar results. E) *C2C12 cell counting*. C2C12 cells were cultured in MSC-DM or in MSC-DMiSK and stimulated or not (vehicle) with 1 µM S1P. After 24 h of culture the cells were trypsinized and counted as described in Materials and Methods Section. The results shown are mean±SEM of at least four independent experiments performed in duplicates. Significance of difference: in C (one-way ANOVA and Newman-Keuls multiple comparison test), *p<0.05 *vs* DM, ^#^p<0.05 *vs* MSC-DM, °p<0.05 *vs* MSC-DMisK; in D (one-way ANOVA and Newman-Keuls multiple comparison test), *p<0.05 *vs* PM, ^#^p<0.05 *vs* MSC-PM, °p<0.05 *vs* MSC-PMisK; in E (Student t test), *p<0.05 *vs* vehicle.

The role played by S1P contained in the conditioned medium from MSCs in the stimulation of myoblast proliferation was confirmed by morphological analysis (Fig. 3A–D). Incubation of C2C12 cells for 24 h with MSC-DM resulted in a significant increase in EdU incorporation and in the immunostaining for Ki67, a nuclear marker protein for cell proliferation, as compared with controls (Fig. 3 A–C). Exposure to MSC-DMiSK blocked by 30–40% the increase in cell proliferation caused by MSC-DM (Fig. 3A–C). As expected, the addition of exogenous S1P to the cells cultured with MSC-DM and MSC-DMiSK further increased C2C12 cell proliferation (Fig. 3A–C, E). Of note, satellite cells showed a similar behavior (Fig. 3D).

Taken together, these results suggest that the synthesis and release of S1P may play an important role in the stimulatory effects of myoblast proliferation mediated by MSC-conditioned medium.

### S1P and VEGF released by MSCs regulate C2C12 cell proliferation

**Figure 4:**
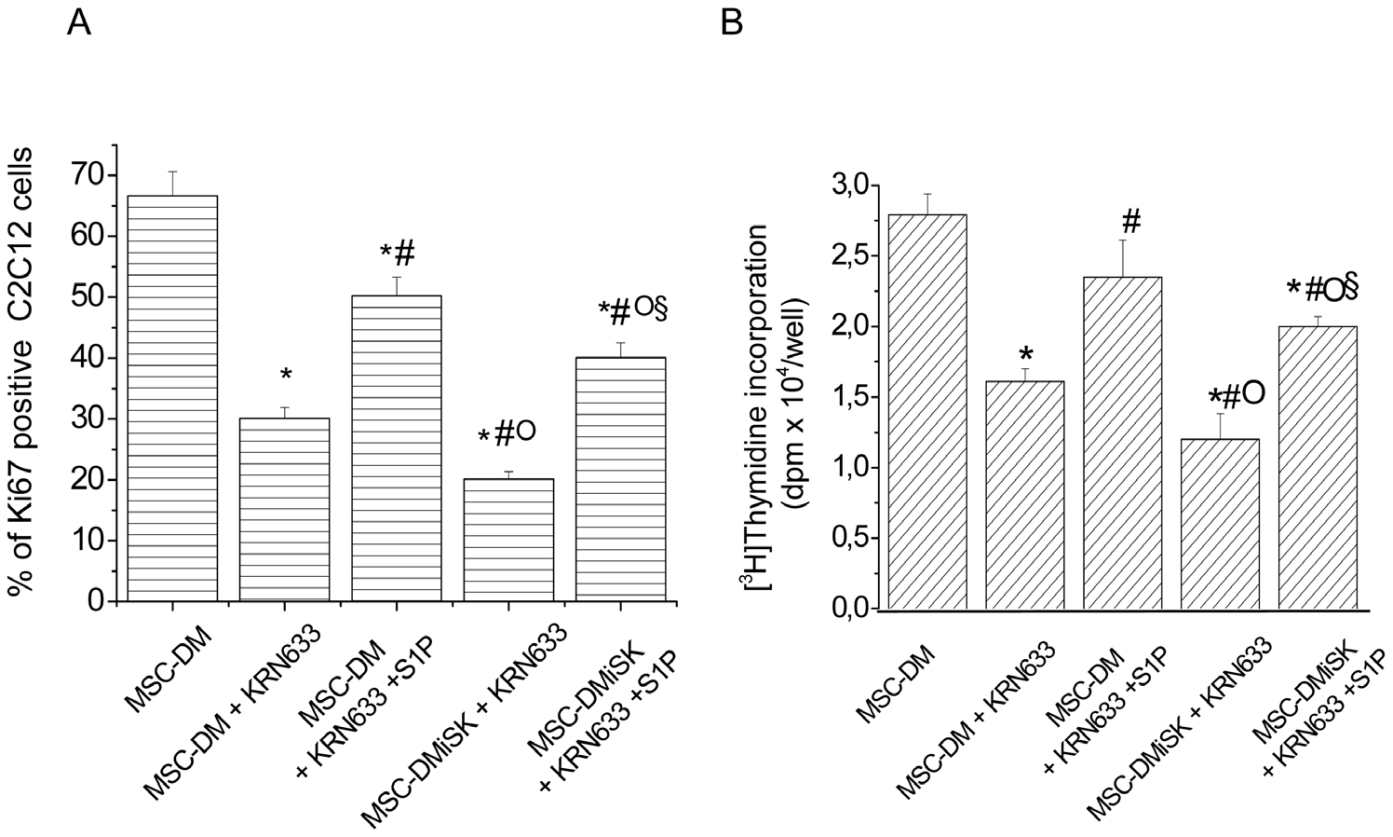
Effect of VEGF signaling inhibition, MSC-secreted S1P and exogenous S1P on C2C12 cell proliferation. C2C12 cells were exposed to MSC-conditioned medium obtained by culturing MSCs for 48 h in differentiation medium (DM) in the absence (MSC-DM) or in the presence of 5 µM iSK (MSC-DMiSK), in the absence and in the presence of KRN633 (170 nM), an ATP competitive inhibitor of VEGFR tyrosine kinase activity, and stimulated or not with 1 µM S1P and then processed for cell proliferation analyses. A) *Confocal immunofluorescence analysis of Ki67 expression in C2C12 cells*. Quantitative analysis of the percentage of Ki67 positive nuclei performed on digitized confocal fluorescent images of the cells immunostained with antibodies against the nuclear protein Ki67. The percentage of Ki67 positive nuclei is expressed as percentage of the total nuclei number (the nuclei were counterstained with PI). The data shown are mean±SEM and represent the results of at least three independent experiments with similar results. B) *[^3^H]thymidine incorporation assay*. The cells were labeled with methyl [^3^H]thymidine and processed as reported in Figure 2B. Significance of difference (one-way ANOVA and Newman-Keuls multiple comparison test) *p<0.05 *vs* MSC-DM, ^#^p<0.05 *vs* MSC-DM+KRN633, °p<0.05 *vs* MSC-DM+KRN633+S1P; ^§^p<0.05 *vs* MSC-DMiSK+KRN633.

**Figure 5:**
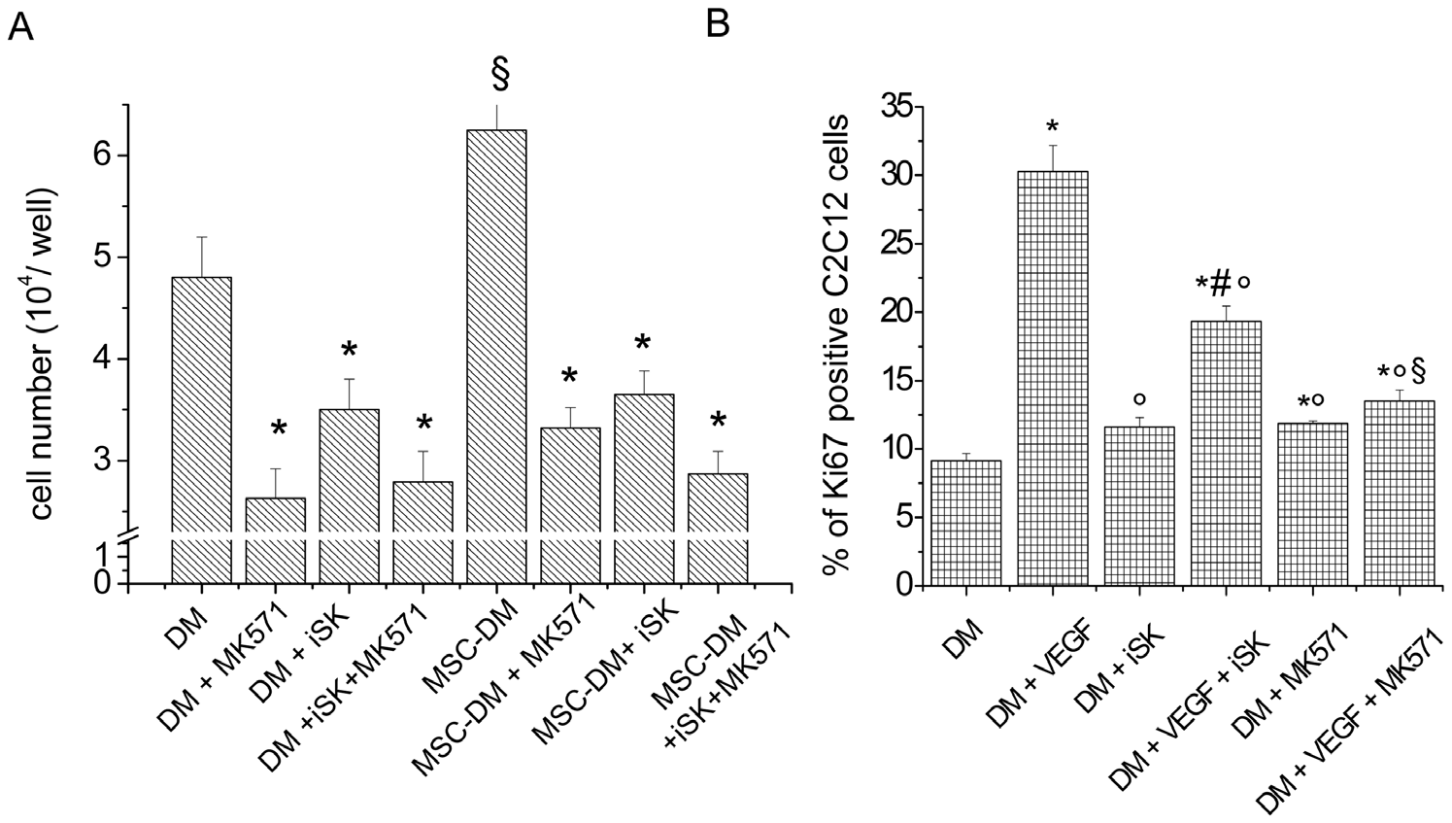
Crosstalk between VEGF and S1P signaling on C2C12 cell proliferation. A) *Cell counting*. C2C12 cells exposed to DM or conditioned medium obtained by culturing MSCs for 48 h in C2C12 differentiation medium (MSC-DM) were treated with 20 µM MK571, a reported S1P transporter inhibitor, or/and with 5 µM iSK for 24 h and trypsinized and then assayed for cell proliferation by cell counting as described in Materials and Methods Section. B) *Confocal immunofluorescence analysis of Ki67 expression in C2C12 cells*. Quantitative analysis of the percentage of Ki67 positive nuclei performed on digitized confocal fluorescent images of C2C12 cells cultured in DM treated or not with 5 µM iSK or with 10 µM MK571, prior the stimulation or not with soluble 2 ng/ml VEGF for 24 h, fixed and then immunostained with antibodies against the nuclear protein Ki67. The percentage of Ki67 positive nuclei is expressed as percentage of the total nuclei number (the nuclei were counterstained with PI). The data shown are mean±SEM and represent the results of at least three independent experiments with similar results. Significance of difference (one-way ANOVA and Newman-Keuls multiple comparison test): in A, *p<0.05 *vs* specific control (DM or MSC-DM), ^§^p<0.05 *vs* DM; in B, *p<0.05 *vs* DM; °p<0.05 *vs* DM+VEGF; ^#^p<0.05 *vs* DM+iSK; ^§^p<0.05 *vs* DM+MK571.

Given that our previous studies have delineated the critical role of VEGF in regulating myoblast cell growth [20] and numerous studies in the literature have shown the existence of a cross-talk between S1P and VEGF signaling [34]–[36], we next analyzed whether these two factors, contained in the conditioned medium obtained from MSCs cultured in DM (MSC-DM), cooperated in the promotion of myoblast proliferation. To this aim, the experiments were repeated in C2C12 cells cultured in the presence of a selective VEGF Receptor (VEGFR) inhibitor, KRN633 (170 nM). It was found that the blocking of VEGF signaling attenuated MSC-DM-mediated cell growth to a similar extent as the reduced level of S1P in the culture medium (approximately by 40% Fig. 4). Notably, co-targeting the VEGF and S1P pathways in C2C12 cells by the treatment of the myoblasts with KRN633 and their exposure to MSC-DMiSK caused an increase of proliferation inhibition of approximately 30% (Fig. 4), indicating that the two signaling pathways could independently affect the regulation of myoblast function. This assumption was confirmed by the data showing that the addition of exogenous S1P was able to prevent the reduction of cell proliferation induced by the inhibition of VEGFR or exposure to MSC-conditioned medium with reduced level of S1P (MSC-DMiSK) or both experimental conditions (MSC-DMiSK+KRN633) (Fig. 4). Finally, in order to evaluate whether the MSC-released factors could regulate C2C12 cell proliferation through the modulation of SphK/S1P signaling of myoblasts, C2C12 cells, incubated in MSC-DM, were treated with 20 µM MK571, a reported S1P transporter inhibitor, or/and with 5 µM iSK and then processed for cell proliferation analyses. Interestingly, the enhanced proliferation induced by the factors released by MSCs, including VEGF, was significantly reduced by the inhibition of S1P transport and slightly more by the combined inhibition of both S1P synthesis and release (Fig. 5A). To confirm a modulation of SphK/S1P axis by VEGF signaling, C2C12 cells in DM were treated with 5 µM iSK or 10 µM MK571 prior the stimulation with 2 ng/ml soluble VEGF and analyzed for Ki67 immunoreactivity. The data reported in Fig. 5B further support the notion that the SphK/S1P axis is a part of VEGF signaling in the control of skeletal muscle cell proliferation.

## Discussion

In the present study we have provided the first experimental evidence that bioactive lipid, S1P, is secreted by MSCs and represents a critical factor by which these cells exert their stimulatory effects on C2C12 myoblast and satellite cell proliferation. Notably, cell proliferation was induced by a concentration of S1P that was in the physiological range [26], [32], [38], [43]. In such a view our data contribute to the understanding of the paracrine effects of MSC-based therapy on the host skeletal muscle and suggest that the release of this sphingolipid by the implanted cells may be a key point for recruiting host myoblasts to participate in the muscle repair. The possible clinical relevance of our findings is highlighted by the following observations: *i)* skeletal muscle is the largest organ of the human body and numerous traumatic injuries and pathologies can affect its functionality [44]; *ii)* the activity of the resident muscle stem cells, satellite cells, representing approximately 2% of the total muscle cells, is severely compromised in degenerative diseases and in volumetric muscle loss [44]–[47]; *iii)* satellite cells, the most obvious cell candidate to be transplanted for muscle regeneration, have some major criticisms due to their heterogeneity [48], [49], loss of stemness after culturing [50] scarce cell survival in the host tissue [51] and the inability to cross the endothelial wall [52], thus restricting their use to local application and the difficulty of obtaining large numbers of cells from a donor. Moreover, the fact that S1P is being recognized as a crucial molecule capable of regulating many fundamental skeletal muscle processes, involving not only the stimulatory action on satellite cell growth [31], [53]–[55], but also resistance to fatigue [56], regulation of muscle contraction [25], protection of the muscle fibers against injury [31], [57] may likely contribute to increase the need of MSC therapy for skeletal muscle disorders.

Our preliminary studies have delineated the essential role of VEGF contained in the conditioned medium from MSCs in regulating C2C12 cell proliferation [20]. Of interest, several levels of cross-talks have been described between VEGF and S1P in many types of normal [34]–[36], [58] and malignant cells [35], [59]–[62]. In particular, VEGF has been shown to regulate S1P receptor expression (S1P_1_) [34], [36], [63], enhance S1P-induced Akt activation [34], [35], [58] and increase intracellular S1P concentration promoting the activation of SphK [35], [59]. Moreover, there is evidence that its receptor, VEGF Receptor-2 (VEGFR-2), forms signaling complex with G-coupled receptor and, more recently, with S1P receptor isoform, S1P_1_, to regulate cell function in some cells [35], [60]. On the other hand, S1P stimulates VEGF expression and secretion in different cell types and phosphorylates and transactivates VEGFR-2 [35], [36], [58], [61]. 

Here, we demonstrated that S1P produced by MSCs exerts its action in part through distinct pathways from VEGF-induced signaling. In fact, our data showed that the simultaneous inhibition of the two pathways evoked a greater reduction of cell proliferation as compared to the single inhibition of VEGF signaling or depletion of S1P from MSC-conditioned medium. Moreover, the addition of exogenous S1P was able to rescue the impairment in cell proliferation caused by VEGFR-2 inhibition. Notably, the data presented in this study, also allow to include the SphK/S1P axis as a potential signaling pathway by which VEGF can regulate skeletal muscle proliferation suggesting the possible interplay between the factors present in the conditioned medium from MSCs. Indeed, inhibition of S1P synthesis or S1P release by C2C12 cells can prevent their proliferative response to MSC-conditioned medium.

Thus, we suggest that the ability of MSCs to secrete different bioactive molecules that can act independently to regulate skeletal myoblast proliferation may represent a critical issue for the success of these cells in tissue repair.

**Figure 6:**
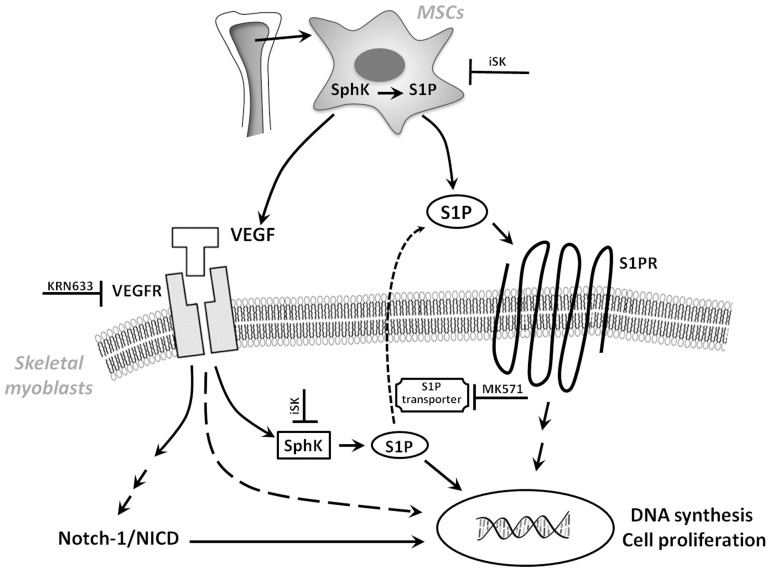
Schematic drawing summarizing the molecular events involved in the promotion of skeletal myoblast proliferation induced by the MSC paracrine action. MSCs, mesenchymal stromal cells; SphK, sphingosine kinase; S1P, sphingosine 1-phosphate; iSK, SphK inhibitor; VEGF, vascular endothelial growth factor; VEGFR, vascular endothelial growth factor receptor; S1PR, S1P receptor subtypes; KRN633, VEGFR inhibitor; MK571, S1P transporter inhibitor; NICD, Notch intracellular domain.

In conclusion our findings have provided novel insights into functional importance of S1P secreted by MSCs in the promotion of skeletal myoblast proliferation (Fig. 6). These findings beside improving our understanding of stem cell biology and action provide a platform for designing new therapeutic approach and experimental model of cell based therapy for improving skeletal muscle regeneration.
